# Epidemiological Study on the Prevalence and Severity of Knee Osteoarthritis in Geriatric Patients With Fragility Fractures of the Hip

**DOI:** 10.7759/cureus.81776

**Published:** 2025-04-05

**Authors:** Keng Pui, Bryan Hon, Michael Shen Xuanrong, Kein Boon Poon

**Affiliations:** 1 Orthopaedic Surgery, Sengkang General Hospital, Singapore, SGP

**Keywords:** fragility hip fracture, kellgren-lawrence grading system, knee osteoarthritis, orthogeriatric, prevalence study

## Abstract

Objective

This study aimed to investigate the prevalence, severity, and association of knee osteoarthritis (OA) in patients with fragility hip fractures in the local population.

Methodology

A cross-sectional study was conducted using retrospective data from 69 patients above the age of 60 admitted to a single orthopedic geriatric trauma center from January to December 2019 for fragility hip fractures.

Results

The prevalence of symptomatic knee OA in the sample was 52% (n = 36) compared to the previous highest known estimate of 11% in the literature (p = 0.00 < 0.05). Among the remaining factors collected, a statistically significant positive linear correlation was found between the severity of the patient’s OA with their BMI (r = 0.238, p = 0.049 < 0.05). The strength of the association was likely weak (0.0 < | r | < 0.3). An association was also found between the ipsilateral knee Kellgren-Lawrence (KL) score and the type of fracture sustained (p= 0.01 < 0.05). Using a multi-nominal logistic regression, the ipsilateral knee KL score was a statistically significant contributor to the model (p = 0.003 < 0.05).

Conclusion

The study showed a 41% higher prevalence of symptomatic knee OA in patients who had sustained hip fractures to those in the general population. An association between the patient’s KL score and the type of fragility hip fracture sustained was also found. Clinically, more emphasis on symptomatic knee OA is necessary in the holistic management of a patient’s fall risk and fragility hip fractures. Further studies should be done to quantify the association between KL scores and the type of hip fracture sustained.

## Introduction

Fragility hip fractures are one of the most common cases of orthopedic admissions in Singapore and continues to be on the rise due to the aging population [1]. Hip fractures pose significant mortality and morbidity risks and financial burden to patients and their caregivers [2]. Considering this, the primary prevention of fragility hip fractures necessitates a thorough assessment of a patient’s fall risks [3]. The demographic profile of a fragility hip fracture patient often precludes a background of polyarticular osteoarthritis (OA).

OA is one of the most prevalent degenerative joint conditions, with the World Health Organization (WHO) estimating symptomatic OA in 9.6% of men and in 18% of women above the age of 60 worldwide [4]. In Singapore, it is estimated that between 4.7% and 11% of the population suffer from symptomatic knee OA [5]. Patients with knee OA have impaired standing and walking balance and a significantly higher fall risk than a similar age- and gender-matched patient without OA [6]. From a biomechanical perspective, knee pain and weakness from OA result in greater postural body sway and center of pressure displacement, which increases the fall risk significantly. The estimated prevalence of falls in older adults was 22% for radiographic knee OA [7], 48% for knee pain, and 48% in symptomatic end-stage knee OA [8].

There have been systematic reviews [9] considering knee pain, impaired balance, muscle weakness, presence of comorbidities, and increasing number of symptomatic joints as risk factors for falls in individuals with knee OA. Their evidence, however, has been conflicting due to the inconsistency of the findings. Limited evidence was found for knee instability, impaired proprioception, and use of walking aids as risk factors for falls.

In prospective population studies, the data are mixed. Selected studies have shown that patients who have symptomatic knee OA that underwent TKR were found to have higher fall risk despite surgery, where fall prevalence remained high at 24-40% even after TKR [8]. There have been several systemic reviews [10] that have shown that TKR improves single limb standing balance and dynamic balance following surgery and positively influences preoperative fallers in becoming postoperative non-fallers. In a more recent systematic review [11] , it was estimated that fall prevalence ranges were reduced from 23-63% to 12-38% for preoperative and postoperative patients, respectively, suggesting that knee OA plays a significant role in fall risks.

Although the link between falls and knee OA is clear, to date, there have been no direct studies demonstrating an association between OA of the knee and fragility fractures of the hip. In our practice, we have observed an extremely high prevalence of severe knee OA in our local hip fracture population. We postulate that the prevalence and severity of knee OA in the local hip fracture population is significantly higher than in the general elderly population and aim to investigate this association in more detail.

## Materials and methods

A cross-sectional study was conducted in Sengkang General Hospital, Department of Orthopedic Surgery, a single orthopedic geriatric trauma center, using retrospective data that were collected and analyzed from the medical records of 99 patients aged above 60 who were admitted between the months of January to December 2019 for hip fractures from falls. Patient data selected for the study were determined based on several inclusion and exclusion criteria (Table 1). Approval from Singhealth Centralised Institutional Review Board (CIRB) was obtained prior to the start of the study (approval no. 2019/2864).

**Table 1:** List of inclusion and exclusion criteria

Inclusion criteria	Exclusion criteria
Age > 60	Pathological fracture of the hip
Admitted for subtrochanteric, inter-trochanteric, or neck or femur fracture	Previous above-knee amputation
Date of admission: January 2019 – December 2019	Previous ipsilateral total knee replacement
Undergone surgical fixation of hip fracture	Terminally ill due to medical comorbidities
	Neuromuscular conditions that affect mobility
	Ongoing work compensation or litigation

Patients with the following factors were excluded: those with concomitant medical conditions that raised their risks of falling or fractures (pathological fractures, terminally ill due to medical comorbidities, neuromuscular conditions affecting mobility), those with previous total knee replacements or above-knee amputations, and those with ongoing work compensation or litigation. After further excluding patients with missing data, the final dataset consisted of 69 cases.

Data collection and definitions

Deidentified patient data were taken from the hospital system's Sunrise Clinical Manager (SCM). A list was generated consisting of all patient's who underwent hip fracture fixation during the period described in the inclusion criteria. For the cases who fit the remaining criteria, perioperative data were collected, which included patient demographic data, biochemical markers, and clinical scores (Visual Analog Score pain score, ambulatory distance over the first three days postoperatively). Patients were further assessed on the presence and severity of any concomitant ipsilateral or contralateral knee osteoarthritis. This was done either via dedicated weight bearing knee X-rays or hip X-rays (with at least two orthogonal views of the knee) in which the ipsilateral knee was included within the view. The radiological images were assessed and graded by two independent assessors according to the Kellgren-Lawrence (KL) grading scheme. Symptomatic knee OA was defined as a KL score of 3 or more.

Patients were also subgrouped based on the type of hip fracture (sub-trochanteric, inter-trochanteric, or femural neck fractures), as well as the type of surgical fixation (hemi-arthroplasty, total hip arthroplasty, cephalomedullary nailing, dynamic hip screw, and cancellous screw fixation) that they had received.

Data analysis

All analyses were performed using IBM SPSS Statistics for Windows, Version 25.0 (released 2017, IBM Corp., Armonk, NY).

## Results

Prevalence of symptomatic OA knee in patients with hip fractures

We hypothesized that individuals with hip fractures have a higher prevalence of symptomatic knee OA (defined as a KL score of 3 or more) than the highest currently known population estimate of 11%. A one-sample binomial test was performed as follows:

We defined the null hypothesis as "the proportion of symptomatic knee OA for the group with hip fractures equals 0.11 or less." The alternative hypothesis was "the proportion of symptomatic knee OA for the group with hip fractures is greater than 0.11." The results of the binomial test are shown in Table 2.

**Table 2:** Binomial test OA: osteoarthritis, KL: Kellgren-Lawrence score

Binomial test						
		Category	N	Observed prop.	Test prop.	Exact Sig. (1-tailed)
Symptomatic OA knee (KL 3-4)	Group 1	Absent	33	0.48	0.11	0.000
	Group 2	Present	36	0.52		
	Total		69	1.00		

Based on the binomial test, there is evidence to suggest that individuals with hip fractures have a higher prevalence of symptomatic knee OA of 52% (n = 36) than the highest known estimate of 11% in the literature (p = 0.00 < 0.05).

Correlations between OA severity and other factors

Further analysis was made into the severity of OA and the remaining relevant factors collected. A Pearson’s correlation test was performed, and the results are shown in Table 3.

**Table 3:** Correlation matrix showing the pearson correlation coefficient and correlation significance OA: osteoarthritis, BMI: body mass index, BMD: bone mineral density, VAS: Visual Analogue Scale * Correlation is significant at the 0.05 level (two-tailed). ** Correlation is significant at the 0.01 level (two-tailed).

		Symptomatic OA knee (KL 3-4)	Age at injury	BMI	Length of stay	HbA1C	BMD femur	VAS post-op day 1	VAS post-op day 2	VAS post-op day 3
Symptomatic OA knee (KL 3-4)	Pearson's correlation	1	0.169	.238^*^	0.109	-0.013	0.178	-0.084	-0.023	0.079
	Sig. (two-tailed)		0.165	0.049	0.374	0.943	0.220	0.506	0.858	0.544
	N	69	69	69	69	33	49	65	65	61
Age at injury	Pearson's correlation	0.169	1	-.267^*^	0.170	-.460^**^	-.321^*^	-0.029	0.106	0.078
	Sig. (two-tailed)	0.165		0.026	0.163	0.007	0.024	0.819	0.401	0.550
	N	69	69	69	69	33	49	65	65	61
BMI	Pearson's correlation	.238^*^	-.267^*^	1	-0.114	0.142	.369^**^	0.238	0.091	0.149
	Sig. (two-tailed)	0.049	0.026		0.353	0.430	0.009	0.057	0.470	0.251
	N	69	69	69	69	33	49	65	65	61
Length of stay	Pearson's correlation	0.109	0.170	-0.114	1	-0.127	-0.103	0.117	0.080	0.102
	Sig. (two-tailed)	0.374	0.163	0.353		0.481	0.481	0.352	0.526	0.435
	N	69	69	69	69	33	49	65	65	61
HbA1C	Pearson's correlation	-0.013	-.460^**^	0.142	-0.127	1	0.165	-0.037	-0.089	-0.123
	Sig. (two-tailed)	0.943	0.007	0.430	0.481		0.451	0.845	0.627	0.523
	N	33	33	33	33	33	23	31	32	29
BMD femur	Pearson's correlation	0.178	-.321^*^	.369^**^	-0.103	0.165	1	0.049	0.046	0.167
	Sig. (two-tailed)	0.220	0.024	0.009	0.481	0.451		0.745	0.766	0.290
	N	49	49	49	49	23	49	46	45	42
VAS post-op day 1	Pearson's correlation	-0.084	-0.029	0.238	0.117	-0.037	0.049	1	.821^**^	.814^**^
	Sig. (two-tailed)	0.506	0.819	0.057	0.352	0.845	0.745		0.000	0.000
	N	65	65	65	65	31	46	65	63	58
VAS post-op day 2	Pearson's correlation	-0.023	0.106	0.091	0.080	-0.089	0.046	.821^**^	1	.773^**^
	Sig. (two-tailed)	0.858	0.401	0.470	0.526	0.627	0.766	0.000		0.000
	N	65	65	65	65	32	45	63	65	59
VAS post-op day 3	Pearson's correlation	0.079	0.078	0.149	0.102	-0.123	0.167	.814^**^	.773^**^	1
	Sig. (two-tailed)	0.544	0.550	0.251	0.435	0.523	0.290	0.000	0.000	
	N	61	61	61	61	29	42	58	59	61

Adjusting for age, we noted on investigation that there was a statistically significant positive linear correlation between the severity of the patient’s OA with their BMI (r = 0.238, p = 0.049 < 0.05).

The strength of the association is likely weak (0.0 < | r | < 0.3). No other statistically significant correlations (p < 0.05) however were found with the remaining factors.

Association between the KL score and type of fracture sustained

We also hypothesized that there was an association between the severity of the individual’s knee OA with the type of hip fracture that was sustained. A chi-square test was performed to evaluate this.

We defined the null hypothesis as "there is no correlation between the KL score of the ipsilateral knee OA to the type of fracture sustained." The alternative hypothesis is defined as "there is a correlation between the KL score of the ipsilateral knee OA to the type of fracture sustained." The results of the chi-square test are shown in Table 4.

**Table 4:** Chi-square test between the Kellgren-Lawrence score of the ipsilateral knee to the type of fracture sustained

Chi-square tests			
	Value	df	Asymptotic significance (two-sided)
Pearson's Chi-square	22.227^a^	6	0.001
Likelihood ratio	23.664	6	0.001
Number of valid cases	69		

Based on the chi-square result, given that the p-value is less than 0.05, we reject the null hypothesis. As such, we concluded that an association was found between the ipsilateral knee KL score and the type of fracture sustained (p = 0.01 < 0.05).

We attempted to investigate this association by performing a multinomial logistic regression (MLR) to ascertain the predictive value of the patient’s KL score, along with age, BMI, BMD hip, and gender on the type of hip fracture sustained. Other variables were omitted due to missing data, which affected the number of valid cases, rendering a total of 49 cases. The full model statistically significantly predicts the type of hip fracture better than the null alone (p = 0.004 < 0.05). Both the Pearson and deviance chi-square statistic likewise suggest that the model fits the data well (Table 5).

**Table 5:** Model fitting information AIC: Akaike information criterion, BIC: Bayesian information criterion

Model fitting information						
Model	Model fitting criteria			Likelihood ratio tests		
	AIC	BIC	-2 log likelihood	Chi-square	df	Sig.
Intercept only	92.279	96.063	88.279			
Final	88.363	118.632	56.363	31.917	14	0.004

Based on the likelihood ratio tests (Table 6), we were able to ascertain that the ipsilateral knee KL score was a statistically significant contribution to the model (p = 0.003 < 0.05). We were unable to derive any useful data from the parameter estimates of the MLR however.

**Table 6:** Likelihood ratio tests AIC: Akaike information criterion, BIC: Bayesian information criterion The chi-square statistic is the difference in -2 log-likelihoods between the final model and a reduced model. The reduced model is formed by omitting an effect from the final model. The null hypothesis is that all parameters of that effect are 0. a. This reduced model is equivalent to the final model because omitting the effect does not increase the degrees of freedom.

Likelihood ratio tests						
Effect	Model fitting criteria			Likelihood ratio tests		
	AIC of the reduced model	BIC of the reduced model	-2 log likelihood of the reduced model	Chi-square	df	Sig.
Intercept	88.363	118.632	56.363^a^	0.000	0	
Age at injury	85.845	112.330	57.845	1.482	2	0.477
BMI	86.568	113.054	58.568	2.206	2	0.332
BMD hip	88.312	114.798	60.312	3.950	2	0.139
Gender	85.706	112.192	57.706	1.343	2	0.511
Ipsilateral knee K/L score	95.869	114.787	75.869	19.506	6	0.003

Findings

Based on the above study, the most important finding was a significantly higher prevalence of symptomatic knee OA in patients who had sustained hip fractures than those in the general population (p < 0.05). The results show a 41% higher incidence of symptomatic OA knee than described in the previous local literature [5]. This study provides further insights into the association between knee OA and fragility hip fractures using a cross-sectional study of patients with traumatic fragility hip fractures in the local context.

On further analysis of the data, we noted a secondary finding in which a significant association was found between the patient’s KL score and the type of fracture sustained (p < 0.05). While previous studies showed that the prevalence of severe knee OA was observed to be higher in certain types of hip fractures [12], our study was able to further suggest that there is significant evidence that the patient’s KL score may predict the type of fragility hip fracture sustained as suggested by the regression study.

A significant positive correlation between a patient’s symptomatic knee OA and their BMI was also shown, which is in keeping with the current literature [13].

## Discussion

Current literature between knee OA and fragility hip fractures

While there are literature works that reported the inverse or an unclear association between the two factors [14-15], a growing number of recent studies worldwide that agree that knee OA may be implicated in fragility hip fractures [16-19]. In Singapore, there is little prevalence data regarding knee OA [20] and none have looked into its prevalence within the population of patients with fragility hip fractures. As such, our study hopes to fulfill this gap in the knowlege within our local context and further corroborate with current findings in regard to the association between knee OA and fragility hip fractures.

Few studies have looked into the association between knee OA and the type of fragility fracture sustained. Davut et al. described a greater prevalence of knee OA in patient's with trochanteric fractures as compared to patients with subtrochanteric fractures [12]. Likewise, our study suggests that there is an association between the severity of knee OA and the type of fragility fracture sustained.

Implications for clinical practice

Notwithstanding the limitations of this study, there remains significant evidence to suggest the prevalence of symptomatic knee OA is indeed higher in groups of patients who have sustained traumatic hip fractures and that the patient’s KL score may predispose patients to certain types of fractures.

Given the considerable clinical, financial, and social consequences of fragility fractures in the elderly along with the association between symptomatic knee OA and fragility fractures of the hip, there is a need to further review the weight to which we view knee OA in the geriatric population.

Clinically, this raises the importance of early identification and management of symptomatic knee OA in this population. The management of knee OA and, in extension, the patient’s fall risk requires a holistic approach. More emphasis may be required to review the severity of a patient’s knee OA during their falls risk assessment either through validated questionnaires in the current literature [5] and potentially screening bilateral knee X-rays for select patients. Interventions such as physical therapy programs may need to be considered earlier given their efficacy, as shown in various studies [21-22], along with the need for adequate pain control. From a surgical perspective, a lower threshold in offering total knee replacements to patients may be considered in patients with symptomatic knee OA. In considering the prevention of falls and its complications in the geriatric population, this may spur further public health studies looking into the cost effectiveness of early surgical intervention for knee OA. Given that several assumptions were made in this study, their limitations need to be considered when interpreting the results of this study.

Limitations to the study

There were several assumptions made during this study which may affect its validity: 1) Given that the study was performed in a single-center orthopaedic geriatric trauma center, which covers North-East Singapore, we assume that the study group is generalizable to the rest of Singapore. 2) For the purposes of the study, symptomatic knee OA was assumed to be a KL score of more than 3. This standard, however, has been used and replicated in other studies [23].

An implication that was identified in the design of the study was the effect of selection bias on the analysis of the results. Given that the sample group was taken from the population who had sustained traumatic hip fractures, this may have skewed the results and hence affected how generalizable the results are.

While there is significant evidence to suggest that the severity of knee OA in a patient is correlated to, and a potential predictor to the type of fracture sustained, we caveat that we were unable to further evaluate and quantify the exact nature of this association due to limitations of the data, likely due to the small sample size, missing data points for different variables collected, and other possible confounders such as the mechanism of injury.

Considering the limitations above, we believe that there is room for further study. Ideally, a case-control study involving multiple orthopedic geriatric trauma centers nationwide would provide further robust data to further improve the generalizability of the study and quantity the predictive value of the patient’s KL score on the type of fracture sustained.

## Conclusions

The prevalence of symptomatic knee OA is 41% higher in patients who sustained hip fractures than those in the general population (p < 0.05) as described in the previous local literature. The study also suggests that there is a significant association and predictive value between the severity of the patient's knee OA and the type of fragility hip fracture sustained.

In light of the findings of this study, the presence of symptomatic knee OA in the geriatric population should not be taken lightly. Its presence should be carefully considered in the geriatric fall risk assessment and simple early interventions such as physiotherapy, and activity modification may be considered. Furthermore, the consideration of a total knee arthroplasty may be broached with the patient at an earlier point of contact.
